# PEANUT: Pathway Enrichment Analysis through Network UTilization

**DOI:** 10.1093/bioinformatics/btaf410

**Published:** 2025-07-21

**Authors:** Yair Pickholz Berliner, Roded Sharan

**Affiliations:** School of Computer Science and AI, Tel Aviv University, Tel Aviv, 69978, Israel; School of Computer Science and AI, Tel Aviv University, Tel Aviv, 69978, Israel

## Abstract

**Summary:**

Pathway enrichment analysis is a fundamental technique in bioinformatics for interpreting gene expression data to pinpoint biological pathways associated with specific conditions or diseases. We introduce Pathway Enrichment Analysis through Network UTilization (PEANUT), a web-based tool for pathway enrichment analysis that enhances traditional pipelines by integrating network propagation computations within a network of protein–protein interactions (PPIs). By diffusing gene expression scores through the PPI network, PEANUT amplifies the signals of connected sets of genes, thereby improving the detection of relevant pathways.

**Availability and implementation:**

The tool is accessible as an open-source web application at https://peanut.cs.tau.ac.il/. The source code is available at https://github.com/Yapibe/PEANUT with a permanent identifier (DOI: https://doi.org/10.5281/zenodo.15184862).

## Graphical abstract

**Figure:**
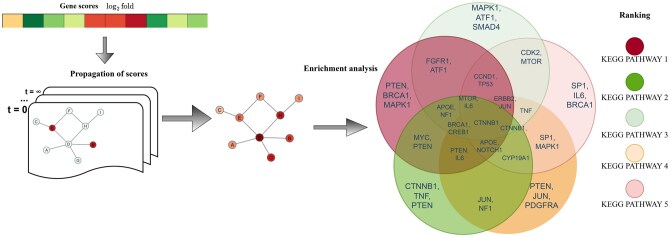

## 1 Introduction

The analysis of gene expression data is a critical step in the elucidation of molecular phenotypes associated with diseases, developmental stages, or responses to treatments. High-throughput technologies such as DNA microarrays and RNA sequencing have generated vast amounts of gene expression profiles, which are publicly available through repositories like the Gene Expression Omnibus (GEO) (Barrett *et al.* 2013). To extract meaningful biological insights from these data, researchers often use pathway enrichment analysis, which identifies biological pathways significantly associated with the observed gene expression changes.

Traditional pathway enrichment methods can be broadly categorized into two types: over-representation analysis and aggregate score approaches (Irizarry *et al.* 2009). Over-representation analysis involves selecting a list of differentially expressed genes (DEGs) and then assessing the over-representation of annotated gene sets among these DEGs. While straightforward, this approach has limitations, including sensitivity to the choice of DEG cutoff and disregard for the relative expression levels of genes just below the threshold.

Aggregate score approaches, such as Gene Set Enrichment Analysis (GSEA) (Subramanian *et al.* 2005), address some of these limitations by considering all genes in the dataset and evaluating the enrichment of gene sets based on the distribution of their members versus that of other genes. GSEA uses a modified Kolmogorov–Smirnov statistic to detect coordinated expression within gene sets. However, GSEA assumes that genes within a gene set are either all up-regulated or all down-regulated, potentially missing gene sets with mixed expression patterns (Saxena *et al.* 2006). Moreover, GSEA does not account for the functional interactions between genes, treating them as independent entities.

Network-based analyses have emerged as powerful tools to incorporate the complex interplay between proteins in biological systems (Cowen *et al.* 2017). By leveraging protein–protein interaction (PPI) networks, these approaches consider the connectivity and relationships between genes, offering a more holistic view of cellular processes. Previous studies have shown that functional genes tend to be proximal to each other in PPI networks and that integrating network information can enhance the identification of disease-associated genes and pathways (Nitsch *et al.* 2009, Jiang *et al.* 2015). This observation motivated the development of the NGSEA network pathway enrichment method (Han *et al.* 2019), which utilizes PPI networks by calculating the absolute average value of the neighbors and adding it to the absolute score of each gene.

Here we introduce Pathway Enrichment Analysis through Network UTilization (PEANUT), a web-based tool that uses network propagation (Cowen *et al.* 2017) to diffuse gene expression scores through a PPI network. This process amplifies the signals of individual genes by considering the expression levels of their network neighbors, thereby capturing the effects of local sub-networks on the biological phenotype. This approach enables the identification of relevant pathways that might be overlooked by methods that consider genes in isolation. In comprehensive evaluations, PEANUT demonstrated improved performance over extant methods in retrieving pathways associated with specific gene expression phenotypes. Furthermore, PEANUT offers an intuitive web interface for uploading gene expression data, customizing analysis parameters, and visualizing results, making it widely accessible.

## 2 Materials and methods

### 2.1 Gene expression profiles and annotated gene sets

To assess the performance of PEANUT in pathway enrichment analysis, we utilized a curated set of gene expression datasets from the “KEGGdzPathwaysGEO” package (Tarca *et al.* 2013). This resource compiles expression data from GEO (Barrett *et al.* 2013) and maps them to specific disease pathways in the KEGG database (Kanehisa *et al.* 2017). In total, we included 24 datasets, each representing a distinct disease and its associated pathway, following established benchmarking practices for pathway enrichment analysis. Of the 24 pathways analyzed, 17 were classified as upregulated and 7 as downregulated, based on the mean of the expression scores for genes within each pathway.

To define the pathway gene sets used for this study, we utilized the C2 curated gene sets from the Molecular Signatures Database (MSigDB) (Subramanian *et al.* 2005, Liberzon *et al.* 2011, 2015). KEGG pathways containing between 15 and 500 genes were retained, resulting in a final set of 427 KEGG pathways for enrichment analysis.

### 2.2 Network propagation

For the network-based analysis, we used a protein network constructed using the ANAT tool (Yosef *et al.* 2011, Signorini *et al.* 2021). To account for both up-regulation and down-regulation of genes, we used the absolute values of the gene expression scores as input to the propagation process. The propagation process can be described using the following equation from the Random Walk with Restart (RWR) method:


(1) $$p_{k}=\alpha p_{0}+(1-\alpha )Wp_{k-1}$$

where $p_{k}$ is the vector of propagated scores at iteration *k*, $p_{0}$ is the initial score vector derived from the absolute gene expression values, *W* is the normalized adjacency matrix of the network, and α is the restart probability that balances between the initial scores and propagated information. We selected α=0.2 as the propagation coefficient to strike a balance between preserving the initial signal and incorporating information from neighboring nodes (Vanunu *et al.* 2010, Charmpi *et al.* 2021). We used a symmetric normalization of *W* as:


(2) $$W=D^{-1/2}AD^{-1/2}$$

where *A* is the adjacency matrix of the network and *D* is the diagonal degree matrix.

### 2.3 Previous methods

To evaluate PEANUT, we compared its performance with traditional Gene Set Enrichment Analysis (GSEA) (Subramanian *et al.* 2005), a replicated version of Network-based Gene Set Enrichment Analysis (NGSEA) (Han *et al.* 2019), and a modified method using absolute scores (ABS GSEA). All methods were implemented using the GSEApy library, ensuring a consistent and reproducible analysis pipeline.

For the primary evaluation, GSEA and ABS GSEA used the full set of genes provided in the dataset. To ensure a fair comparison with PEANUT and NGSEA, which rely on gene scores derived from the intersection of genes present in both the dataset and the network, we also ran an additional analysis where GSEA and ABS GSEA were restricted to this intersection. This secondary evaluation served to standardize the input across all methods.

#### 2.3.1 GSEA

We used the original gene expression scores directly from the datasets without any modification. These scores were ranked and fed into the GSEA PreRank tool to assess pathway enrichment using KEGG pathways.

#### 2.3.2 ABS GSEA

As an additional control method, we replaced the original gene expression scores with their absolute values before ranking them. These ranked absolute scores were then input into the GSEA PreRank tool for pathway enrichment analysis.

#### 2.3.3 NGSEA

Since we were unable to replicate the exact results from the original NGSEA paper (Han *et al.* 2019), and no source code or list of input pathways was provided, we reimplemented NGSEA based on its published description. Specifically, for each gene *i*, we assigned the absolute expression value and added the average absolute expression of its immediate network neighbors:


(3) $$NS_{i}=|x_{i}|+\frac{1}{n_{i}}\sum_{j=1}^{n_{i}}|x_{j}|$$

where $n_{i}$ is the number of neighbors of gene *i*, and $x_{j}$ are the expression values of those neighbors.

### 2.4 PEANUT

We developed a novel pathway enrichment analysis method that combines network propagation with a series of advanced statistical tests to identify biologically relevant pathways associated with the propagated gene expression scores.

After network propagation, we first applied the Kolmogorov–Smirnov (K–S) test to compare the distribution of propagated scores within each pathway to the background distribution of scores outside the pathway. For pathways that were significant in the K–S test, we conducted the Mann–Whitney U test as an additional validation step, comparing the ranks of pathway gene scores with those of background genes. Finally, we performed a permutation test with 10 000 iterations to empirically evaluate the significance of the observed pathway scores. This test generated a null distribution by randomly sampling genes, allowing us to compute empirical *P*-values based on the rank of the observed pathway scores within this distribution. We chose 10 000 permutations as a balance between achieving sufficient statistical power for robust *P*-value estimation and maintaining reasonable computational efficiency.

To adjust for multiple comparisons across all tests, we applied the Benjamini-Hochberg correction (Benjamini and Hochberg 1995) after each stage (K–S, Mann–Whitney U, and permutation tests), controlling the false discovery rate (FDR) and identifying pathways with an adjusted *P*-value threshold of .05 as significant.

## 3 Results

### 3.1 Performance evaluation

The performance of PEANUT was evaluated against GSEA, NGSEA, and ABS GSEA using 24 disease-associated gene expression datasets from the “KEGGdzPathwaysGEO” package (Tarca *et al.* 2013). These datasets contained ${\;\text{log}\;}_{2}\text{fold}$ scores as input. The distributions of ranks assigned by each method to the true associated pathways were compared to assess the significance of differences between each pair of methods. In this context, “rank” refers to the position of the true associated pathway in the list of all pathways sorted by their enrichment significance, with lower ranks indicating higher enrichment.

**Figure 1.**
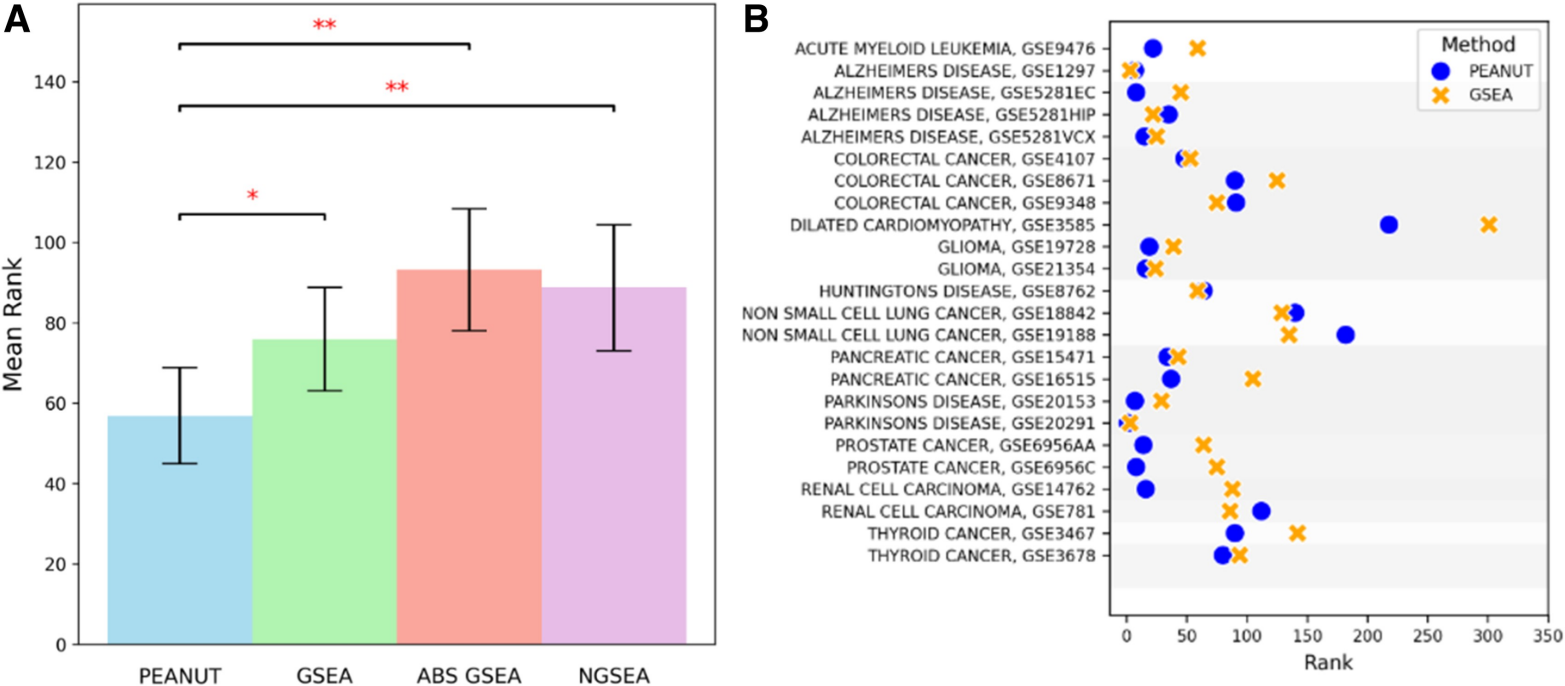
(A) Histogram comparing rank distributions of all methods (PEANUT, ABS GSEA, GSEA, and NGSEA) across the datasets when using the full dataset. Lower ranks indicate better performance. Statistical significance of differences in rank distributions was assessed using the Wilcoxon signed-rank test (*P<.05, **P<.01). (B) Scatter plot comparing the ranks of the associated pathways assigned by GSEA (second best method) and PEANUT across 24 datasets when using the full dataset. Each point represents a dataset, with PEANUT achieving consistently lower ranks.

As shown in Fig. 1A, PEANUT consistently achieved lower ranks for the associated pathways compared to GSEA across the datasets when using the full set of genes in the dataset. PEANUT ranked better than GSEA in 17 of the 24 pathways, with an average rank difference of 34.3 in those pathways. Conversely, in the seven pathways where GSEA outperformed PEANUT, the average rank difference was only 16.3. When directly compared to GSEA, as illustrated in Fig. 1B, PEANUT demonstrated a statistically significant improvement (*P*<.02), with a mean rank difference of 19.1 in favor of PEANUT. Similarly, PEANUT achieved significantly better ranks than NGSEA (*P*<.003), with a mean rank difference of 31.9, and outperformed ABS GSEA (*P*<.005), with a mean rank difference of 36.4. These results emphasize the advantage of incorporating network propagation in pathway enrichment analysis, as implemented by PEANUT.

While both NGSEA and PEANUT incorporate network information, their approaches are fundamentally different. NGSEA smooths scores using local neighbor averaging, whereas PEANUT performs global network propagation, diffusing information across the entire network. This allows PEANUT to amplify coordinated signals from interconnected sub-networks, even when some relevant genes have weak or missing scores. As a result, PEANUT is better equipped to identify pathways impacted by distributed but functionally related genes.

Unlike the original NGSEA paper, which reported NGSEA outperforming GSEA, we observed the opposite trend in our implementation. This discrepancy may stem from several factors. First, the original NGSEA study used a curated subset of 276 KEGG pathways, which was not made publicly available, while our analysis used 427 KEGG pathways from MSigDB (Liberzon *et al.* 2011) that passed standard size filtering. The difference in pathway coverage may affect performance: using fewer pathways can reduce the chance of false positives and may inflate apparent accuracy. Second, as the NGSEA source code and full experimental setup were not available, we could not replicate their analysis exactly. These differences likely contribute to the observed divergence in performance trends.

In addition to ranking accuracy, the significance level assigned by each method to the true pathways was evaluated. For PEANUT, a pathway was considered significant if it had both a significant Kolmogorov-Smirnov (KS) *P*-value and a Mann-Whitney U (MW) *P*-value. For the GSEAPy-based methods (GSEA, ABS GSEA, and NGSEA), significance was determined using the FDR-adjusted *q*-value calculated by GSEAPy. Using the full dataset, PEANUT identified 58% of pathways as significant, compared to 46% for GSEA, 0.8% for ABS GSEA, and 21% for NGSEA, highlighting its enhanced sensitivity in identifying biologically relevant pathways.

To assess the impact of restricting methods to the intersection of genes present in both the dataset and the network, we conducted a secondary analysis. Under this condition, PEANUT maintained its performance advantage, achieving statistically significant improvements over GSEA (*P*<.01, mean rank difference = 21.7) and ABS GSEA (*P*<.002, mean rank difference = 38.3).

### 3.2 Development of the PEANUT web server

To facilitate access and ease of use, we developed PEANUT as a user-friendly web server available at https://peanut.cs.tau.ac.il/. The web interface allows researchers to perform network-based pathway enrichment analysis without requiring extensive computational expertise. Users can upload their pre-ranked gene lists in the .rnk or .xlsx file formats, which are supported as standard inputs for the platform. The platform supports customizable parameters, including the propagation coefficient (α), number of permutations, the choice of PPI network (e.g. ANAT), and the selection of pathway databases for analysis.

To address user needs, we have included detailed help features accessible via information (“?”) buttons next to each parameter. These provide explanations of terms such as the propagation coefficient and guidelines for selecting pathway databases. Additionally, we provide sample data files that users can download and test on the platform, ensuring they can familiarize themselves with the workflow before analyzing their own data.

PEANUT supports various pathway databases, including the Kyoto Encyclopedia of Genes and Genomes (KEGG), which provides a comprehensive collection of manually curated pathway maps. The server allows simultaneous execution of traditional GSEA and network-based PEANUT analyses, enabling direct comparisons between results. It generates comprehensive output that includes ranked lists of enriched pathways, statistical significance measures such as Enrichment Score, and False Discovery Rate, along with interactive enrichment plots for visual interpretation of results.

The PEANUT tool is available at https://github.com/Yapibe/PEANUT and has been assigned a Zenodo DOI: https://doi.org/10.5281/zenodo.15184862.

## 4 Conclusions

PEANUT represents a significant advancement in pathway enrichment analysis by integrating network propagation with a robust statistical framework. By leveraging network propagation, PEANUT amplifies biological signals, enabling the identification of disease-relevant pathways with higher accuracy and significance compared to traditional methods like GSEA and NGSEA. Our results demonstrate that PEANUT consistently outperforms these alternative methods in ranking associated pathways, with statistically significant improvements in performance metrics such as rank and significance. Furthermore, PEANUT’s ability to effectively prioritize related pathways underscores its utility in providing a more comprehensive understanding of biological processes, even in cases where the associated pathway is not prominently ranked.

The development of the PEANUT web server further enhances its accessibility, allowing researchers to easily customize parameters and compare results across methods. Overall, PEANUT’s combination of methodological innovation and user-friendly implementation positions it as a powerful tool for pathway enrichment studies, with the potential to drive deeper insights into complex biological systems.
